# Core-Shell Nano-Antenna Configurations for Array Formation with More Stability Having Conventional and Non-Conventional Directivity and Propagation Behavior

**DOI:** 10.3390/nano11010099

**Published:** 2021-01-04

**Authors:** Qaisar Hayat, Junping Geng, Xianling Liang, Ronghong Jin, Sami Ur Rehman, Chong He, Haobo Wu, Hamza Nawaz

**Affiliations:** Department of Electronics Engineering, Shanghai Jiao Tong University, Shanghai 200240, China; qaisarhayat@sjtu.edu.cn (Q.H.); liangxl@sjtu.edu.cn (X.L.); rhjin@sjtu.edu.cn (R.J.); samiurrehman@sjtu.edu.cn (S.U.R.); hechong1002@163.com (C.H.); haobowu@sjtu.edu.cn (H.W.); hamza_nawaz@sjtu.edu.cn (H.N.)

**Keywords:** Terahertz photonics, optical array configuration, nano-antenna, core-shell antenna structure, plasmonic

## Abstract

The enhancement of optical characteristics at optical frequencies deviates with the choice of the arrangement of core-shell nanoparticles and their environment. Likewise, the arrangements of core-shell nanoparticles in the air over a substrate or in liquid solution makes them unstable in the atmosphere. This article suggests designing a configuration of an active spherical coated nanoparticle antenna and its extended array in the presence of a passive dielectric, which is proposed to be extendable to construct larger arrays. The issue of instability in the core-shell nanoantenna array models is solved here by inserting the passive dielectric. In addition to this, the inclusion of a dielectric in the array model reports a different directivity behaviour than the conventional array models. We found at first that the combination model of the active coated nanoparticle and passive sphere at the resonant frequency can excite a stronger field with a rotated polarization direction and a propagation direction different from the incident plane-wave. Furthermore, the extended 2D array also rotates the polarization direction and propagation direction for the vertical incident plane-wave. The radiation beam operates strong multipoles in the 2D array plane at resonant frequency (behaving non-conventionally). Nevertheless, it forms a clear main beam in the incident direction when it deviates from the resonance frequency (behaving conventionally). The proposed array model may have possible applications in nano-amplifiers, nano-sensors and other integrated optics.

## 1. Introduction

The plasmonic nano-antennas or the core-shell nanoparticles have remained a subject of research due to their potential applications in many areas such as solar energy harvesting, medicine, spectroscopy, biosensing, nano-scale lithography, microscopy, data storage, on-chip surface plasmon generation, nano-amplifiers and nano-sensor applications [1,2,3,4].

Recently, different coated nanoparticle (CNP) structures were presented, including magneto-electric core-shell nanoparticles [5], gold nanoparticle-based core-shell [6], 3-D arrays of Au-SiO_2_ core-shell nanoparticle [7], metal-semiconductor [8], and metal-dielectric [9]. Normally these different reported CNPs are the passive CNPs and have high losses at an optical regime that limits their use in the amplification [10,11]. However, the addition of some gain material to the CNPs compensates these losses, which makes them Active Coated Nanoparticles (A-CNPs). Multiple models are presented to describe active nanoparticles [11,12,13,14]. This inclusion of gain in passive material significantly enhances the resonance characteristics as well as narrowing the response to 1 THz [15,16,17]. Besides these enhancements in optical characteristics, there are still several limitations in single-antenna systems that motivate the development of nano-antenna arrays, such as the small size of a single nano-antenna, which always suffers from inadequate available output power, and the challenges in managing its radiation pattern in the far-field. In addition to this, due to the recent interest of the modern age in monitoring the directivity of the field’s radiated by nano-antenna structures, a specific design is required for enhancement of the emitted far-field directivity [18,19,20]. Nano-antenna array configuration is a good method to enhance directivity, but still has the reported problem of the instability of placing bare CNPs.

The core-shell nano-antennas presented, experimentally and theoretically, to date, are in liquid-solution form or suspended in the air placed on the substrates, which makes them unstable in the environment. Moreover, there are still difficulties in the regular arrangement of core-shell nanoparticles that can help in the enhancement of optical characteristics, along with a stable environment for these core-shell nanoparticle antennas [5,9,14,21,22]. In our previous work, we presented a Plasmonic-Induced Polarization Rotator (PI-PR) comprising a single/two active core-shell CNPs capable of polarization rotation with the help of passive CNPs [23]. We believe that an extended array model, based on the PI-PR model, can be achieved, which can solve the problem of instability in these passive and active CNP models.

This paper provides a mechanism of modelling an array configuration and its extended array model from an individual active spherical coated nano-particle (A-CNP) to improve the directivity and radiation performances in a more stable environment. Introducing the passive dielectric between the identical active CNP resonators can solve the problems of instability offered by these core-shell nanoparticle arrays. Besides this, the behavior and performance of this array model are different from the conventional array models at the resonance frequency. We observed that this array model radiates in a multipolar mode at resonance frequency but has stronger directivity at nearby frequencies at a 0.01 THz and 0.008 THz difference from resonance frequency (conventional directivity). Normally, the conventional radiation beam of an antenna or its array generates on the broad side or the end fire [24]. However, the observed radiation beam in this article radiates in a multipolar mode at resonance frequency, i.e., is totally different from conventional antennas. In addition to this, the conventional broad side beam is also formed at very close resonance frequencies. Similarly, the direction of the electric field of scattering radiation is perpendicular to the incident electric field direction, that is, the polarization of the array model changes its direction by 90° with incident plane-wave polarization. This paper analyzes and compares passive inclusion configurations as well as their extended array models with the passive excluding configurations constituted by the arrangement of A-CNPs. The inclusion of a stable and larger-sized passive medium supports the A-CNPs on a substrate as well as storing the energy when an A-CNP resonates. The array with passive can be easy to realize and increase the directivity, but limits the SCS by a small value due to energy storing in the passive silica. We also suggest that the passive medium may enclose the whole CNP array in future.

Section 2 describes the materials and the plane-wave excitation used in these formulations and depicts the basic model formulation used to design two possible configurations, I and II, for scheming an array. Section 3 elaborates the simulation results of two configurations, whereas Section 4 gives the design of two models based on the two configurations. Finally, Section 5 gives the simulation results of the proposed extended array designed from the two configurations and their models.

## 2. Materials, Design and Analysis

A computational electromagnetic simulation tool of CST Microwave Studio, which solves Maxwell’s equations using the frequency-domain method, is used here to analyze the problem of designing an active spherical coated nanoparticle (CNP) model. This paper shows the construction of the CNP model to design two possible array configurations in a systematic way for optical characteristics enhancement. Finally, we construct possible array models based on these two configurations in the last section. The metallic material silver used here obeys the size effect Drude model at optical wavelengths between 350 and 800 nm; a detailed explanation and calculation is presented in reference [17,25]. Similarly, the gain medium inserted here, also termed the active core, includes the rare-earth-doped ${\mathrm{SiO}}_{2}$, which is present in references [14,17,26]. We use similar gain media values for spherical active CNPs, as shown in reference [14].

### 2.1. Planewave Excitation

Scattering Cross-Section (SCS) and Absorption Cross-Section (ACS) can characterize the behavior of the core-shell nano-particle excited by electromagnetic plane-wave. SCS is the integrated power enclosed in the scattered field normalized by the irradiance of the incident field, whereas ACS is the net power flux through a surface surrounding the concentric shells normalized by the incident field irradiance. The scattered and absorbed power can be expressed by the following expression via Poynting’s theorem

(1) $$P_{scat}=Re\left\{\frac{1}{2}\iint_{s}^{\;}\left[{\vec{E}}_{s}\times {\vec{H}}_{s}^{*}\right]\cdot \hat{n}dS\right\}$$

(2) $$P_{abs}=-Re\left\{\frac{1}{2}\iint_{s}^{\;}\left[{\vec{E}}_{tot}\times {\vec{H}}_{tot}^{*}\right]\cdot \hat{n}dS\right\}$$

where “*S*” is the surface enclosing the particle and $\hat{n}$ is a unit vector pointing outward from the surface. The total RCS, ACS and ECS can be defined by the ratio of radiated power with incidence irradiance [14].

(3) $$\sigma_{scat}=\frac{P_{scat}}{I_{inc}}$$

(4) $$\sigma_{abs}=\frac{P_{abs}}{I_{inc}}$$

The values of $\sigma_{scat}$ and $\sigma_{abs}$ are readily calculated with the CST post-processing tools.

### 2.2. Model of Active Spherical Coated Nanoparticles

**Figure 1:**
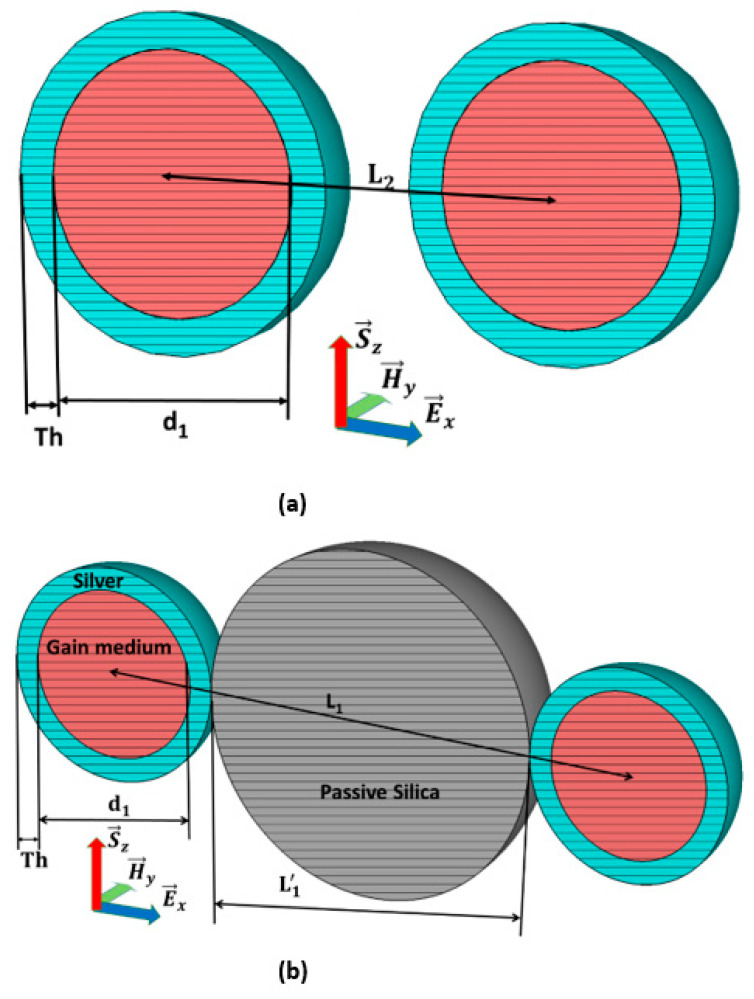
Configurations of dual active coated nanoparticle (CNP) model: (**a**). Two spherical active CNPs constructs’ array configuration-I and (**b**) Array Configuration-II in the presence of passive silica between two CNPs.

Coated nano-particle (CNP) is the basic radiated element, in which the shell is silver, and the silica doped with Er^3+^ fills the core. The coated nano-particle model is depicted in Figure 1, where silver fills the shell of thickness Th while active silica occupies its core with a diameter $d_{1}$, and hence it constructs an active spherical coated nanoparticle (CNP). The basic CNP model creates two configurations, as given in the following.

### 2.3. The Configuration-I from the Basic Model of the Double CNPs

Figure 1b shows the basic model of configuration-I in the form of two particles’ configuration, where two CNPs are placed at 0.72λ away from their centers. The parameters set $d_{1}=46\;\mathrm{nm}$ and Th=6 nm here construct the CNP particle with the initial interval $L_{2}=0.72\lambda$, while the CNP pair is in the vacuum. This model is convenient to extend to the CNP array.

### 2.4. The Configuration-II from Double CNPs and Passive Nano-Spheres

The simplified model of configuration-II of the mixed nanoparticles is given in Figure 1a; the passive sphere is sandwiched between two active CNPs. The CNP is the same as in configuration-I, and the passive silica of diameter is $L_{1}^{\prime }=313\;\mathrm{nm},$ supplanting the space between the CNPs to maintain the $\;L_{1}=0.742\lambda$ spacing between the centers of CNPs. This simplified model is the basic unit to fix the uniform distribution of the extended CNP array.

## 3. Analysis of 2-CNP Array Unit Configurations

**Figure 2:**
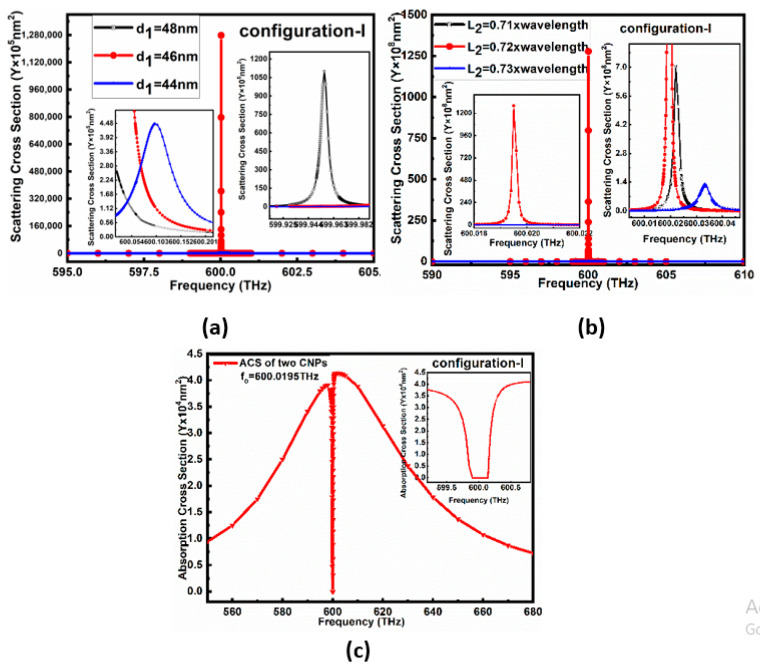
shows scattering cross-section (SCS) and absorption cross-section (ACS) for configuration I varies with different size: (**a**) SCS varies with the core diameter *d*_1_ of CNP for configuration-I. (**b**) SCS ($127.79\times 10^{9}\;{\mathrm{nm}}^{2}$ ) varies with the interval *L*_2_ between two CNPs in case of configuration-I, (**c**) ACS ($4.5\times 10^{4\;}{\mathrm{nm}}^{2}$) for configuration-I corresponding to $L_{2}=0.72\lambda$.

The first non-canonical geometry, active spherical Coated Nanoparticle (CNP), considered here, consists of an active SiO_2_ core of diameter $d_{1}=46\;\mathrm{nm}$ surrounded by a silver shell (Drude model) of thickness Th=6 nm, as illustrated in Figure 2a. The silver thickness choice corresponds to the cases in [14,17,23] for the active spherical CNP. However, the performance of CNPs throughout the optical regime is optimizable with the variations in both the radius and core material.

### 3.1. ${\vec{E}}_{x}$ Polarization Plane Wave Incident

The ${\vec{E}}_{x}$ polarized plane-wave of amplitude $E_{0}=1.0\;V/m$ propagating along *z*-axis excites the whole basic configurations, where the electric field is parallel to the *x*-axis. The plane-wave can be expressed as

(5) $${\vec{E}}_{\;}=\hat{x}E_{0}cos\left(\omega t+kx\right)$$

The selection of frequency interval is in accordance with our prediction that the resonance frequency is near 600 THz or 500 nm wavelength. The permittivity of the silver shell and gain medium core comprises both real and imaginary components. The real components of permittivity contribute the displacement currents while the imaginary components contribute to conduction currents. Here, we have selected opposite signs of real parts of the permittivity of a silver shell and gain media core to attain an electrically small resonator. Correspondingly, the imaginary part of permittivity for the gain core near the resonance frequency is selected as negative, while for the shell it is selected as positive in the same region, to dominate the conduction current. Consequently, the opposite signs of the imaginary parts of permittivity in the core and shell form the current densities in the opposite direction in the core to the shell. This enhanced current distribution increases the radiated power.

The simulation outcomes of configuration I for similar parameters to the single CNP proved that the resonating frequency of the gain media is at a lower frequency than that of the desired frequency. Therefore, the diameters $d_{1}$ of gain media, in the core of the CNP for configuration I, were first optimized, as shown from the scattering cross-section peaks in Figure 2a. It is clear from Figure 2a that the resonating peaks in the case of $d_{1}=46\;\mathrm{nm}$ have high dominancy over other values of $d_{1}$ near the anticipated resonance frequency.

In the case of configuration-I for *L*_2_ optimization, the black, red and blue lines in Figure 2b represent the SCS peaks for $L_{2}=0.71\lambda$, $L_{2}=0.72\lambda$, and $L_{2}=0.73\lambda$, respectively. The maximum SCS peak reported in Figure 2b is $127.79\times 10^{9}{\;\mathrm{nm}}^{2}$ at $f_{o}=600.0195\;\mathrm{THz}$ for configuration-I. In addition to this, the minimum ACS for configuration I is $4.5\times 10^{4}\;{\mathrm{nm}}^{2}$, below the background values, as reported in Figure 2c.

**Figure 3:**
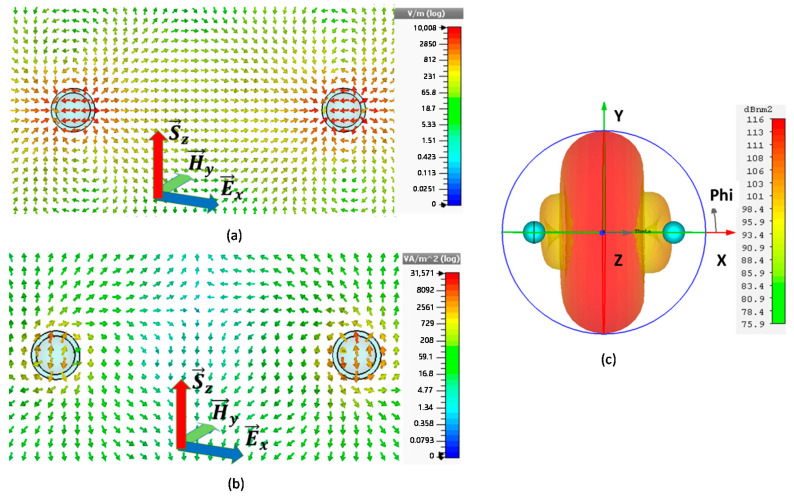
Field distribution of A-CNPs configuration I with maximum SCS peak at f=600.0195 THz: (**a**) electric field distribution, (**b**) power flow in xoz plane. (**c**) far-field pattern.

Figure 3 explains the electric field and power flow of array configuration-I. At the resonance state, f = 600.0195 THz, the electric field reported in Figure 3a looks like two dipoles with the same phase, and the maximum E-field value is $1.0008\times 10^{4}\;V/m$. Figure 3b shows the view of its power flow in XOZ cut plane, and its peak is $3.1571\times 10^{4}\;\mathrm{VA}/m^{2}$. From the arrows’ direction, it is observable that the power is pointing out from the gain media center and flows in the YOZ plane. The far-field pattern for configuration-I at resonant frequency 600.0195 THz is shown in Figure 3c, which is the pattern of the binary array with equal amplitude and the same phase, and it displays $116{\;\mathrm{dBnm}}^{2}$ maximum RCS.

**Figure 4:**
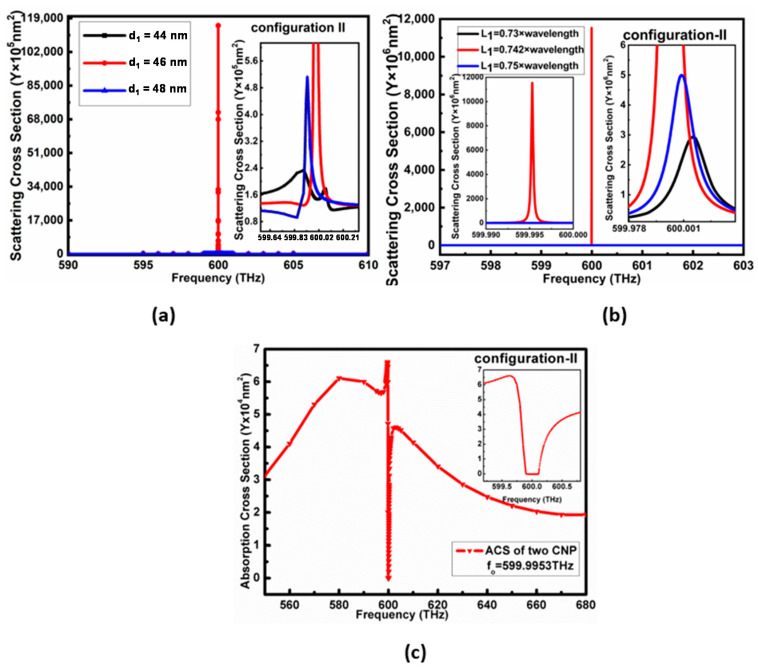
shows optimization results for configuration 2. (**a**) shows optimization peaks of $d_{1}$, (**b**) denotes the optimization of $L_{1}$ for configuration-II; here, SCS $\left(11.53\times 10^{9\;}{\mathrm{nm}}^{2}\right)$ peaks shown by black, red and blue line represent SCS for $L_{1}=0.73\lambda \;\mathrm{nm}$, $L_{1}=0.742\lambda \;\mathrm{nm}$, and $L_{1}=0.75\lambda \;\mathrm{nm}$ passive spacing, respectively. (**c**) shows ACS ($6.5\times 10^{4}{\;\mathrm{nm}}^{2}$) for configuration-II.

The simulation outcomes of configuration II for the similar parameters to single CNP proved that the resonating frequency of gain media is at a lower frequency than the desired frequency. Therefore, the diameters $d_{1}$ of gain media in the core of CNP for configuration II were first optimized, as shown from the scattering cross-section peaks in Figure 4a. It is clear that the resonating peaks in the case of $d_{1}^{\;}=46\;\mathrm{nm}$ for configuration II have high dominancy over other values of $d_{1}$ near the anticipated resonance frequency.

The optimized SCS peaks in configuration-II are shown in Figure 4b for *L*_1_ optimization; the black, red and blue lines represent the SCS peaks for $L_{1}=0.73\lambda$, $L_{1}=0.742\lambda$, and $L_{1}=0.75\lambda$, respectively. The maximum SCS peak shown in Figure 4b for configuration-II is $SCS=11.53\times 10^{9}{\;\mathrm{nm}}^{2}$ higher than the ground value at the frequency $f_{o}=599.9953\;\mathrm{THz}$. In addition to this, the minimum of ACS for configuration II is $6.5\times 10^{4}\;{\mathrm{nm}}^{2}$, below the background values, as reported in Figure 4c.

**Figure 5:**
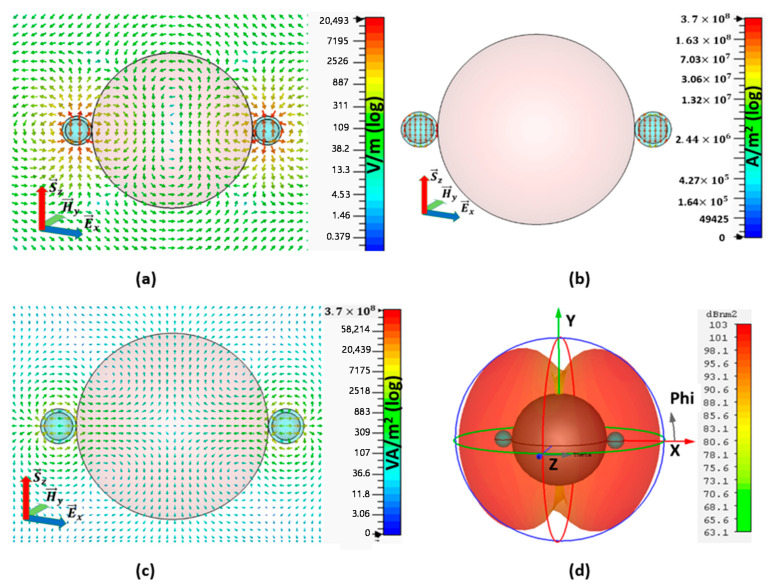
Field distribution of A-CNPs configuration II with maximum SCS peak at *f* = 599.9953 THz: (**a**) electric field distribution for Configuration II. (**b**) current density distribution. (**c**) power flow and (**d**) far-field pattern for configuration II at the resonance frequency.

The electric field, current density, power flow and far-field pattern of the array configuration-II at the resonant frequency $f_{o}=599.9953\;\mathrm{THz}$ are given in Figure 5. At the resonance state, the electric field distribution in XOZ cut plane is rotationally symmetrical, and the local E-field around the active CNP looks like a dipole. Nevertheless, the direction of the E-field of these two local regions is opposite to that Figure 5a, and the maximum amplitude of E-field for configuration-II is 20493 V/m. The reason for the rotational symmetrical E-field distribution is that the plane wave is refracted by the passive sphere at first, and then constructs the rotational electric field in the dielectric sphere, which excited the opposite TM_11_ modes in these two active CNPs, which are both CNPs resonates at 180° phase difference from each other, as shown in Figure 5a.

The opposite symmetrical current density distributions are shown in Figure 5b, and the maximum current density is $3.77\times 10^{8}\;A/m^{2}$. Here, current direction is clearly shown to be perpendicular to the incident electric field direction and supports the arguments of electric field rotation. In addition to this, Figure 5c shows the XZ cut plane view of the power flow for configuration II, which is $1.66\;V\times 10^{5}\;A/m^{2}$. Here, the power is pointing out from the gain media and flows along the *x*-axis which is perpendicular to the original plane-wave direction.

The far-field pattern for configuration-II in Figure 5d reports $116{\;\mathrm{dBnm}}^{2}$ RCS; here, the flow of energy represented by the dipolar form for configuration-II is along the *y*-axis, which verifies the power-scattering direction as detailed in Figure 5c. Different from the pattern of the binary array with equal amplitude and the same phase in Figure 3c, here the far-field pattern for configuration-II in Figure 5d is just like the pattern of the binary array with equal amplitude and inverse phase.

### 3.2. ${\vec{E}}_{y}$ Polarized Configuration II

**Figure 6:**
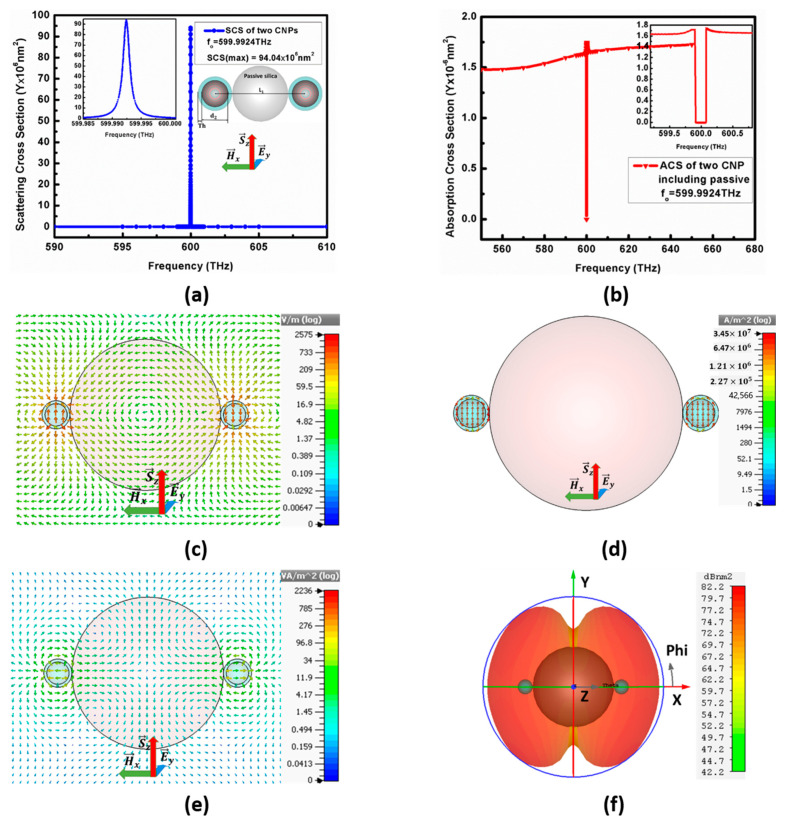
Represents (**a**) SCS of configuration-II, (**b**) ACS, (**c**) electric field spectrum, (**d**) current density (**e**) power flow and (**f**) far-field pattern for configuration II.

The ${\vec{E}}_{y}$ polarized plane-wave propagating along the *z*-axis excites the optimized structure here to observe the variations in optical characteristics. The maximum SCS shown in Figure 6a for configuration-II is $94.4\times 10^{6\;}{\mathrm{nm}}^{2}$ at resonant frequency $f_{o}=599.9924\;\mathrm{THz}$, whereas the minimum ACS for this configuration is $1.6\times 10^{-6}\;{\mathrm{nm}}^{2}$ below the background value, as shown in Figure 6b. Figure 6c,d reports the electric field as E=2575 V/m and the electric current density is $3.45\times 10^{7}\;A/m^{2}$.

The radiated power for configuration I is $2236\;\mathrm{VA}/m^{2}$, which orients from centers of resonating CNPs and flows along the *x*-axis that is at 90° with the incident plane-wave direction, as illustrated by the XZ cut-plane view in Figure 6e. The far-field patterns in Figure 6f at the resonant state show a maximum $82.2\;{\mathrm{dBnm}}^{2}$ RCS for configuration II. Similar to a result in Figure 5d, here the far-field pattern is also like the pattern of the binary array, with equal amplitude and inverse phase. Furthermore, the far-field pattern confirms the direction of power flow along the *x*-axis, as demonstrated in Figure 6e.

### 3.3. ${\vec{E}}_{z}$ Polarized Plane-Wave Excitation Propagating along the x-Axis

**Figure 7:**
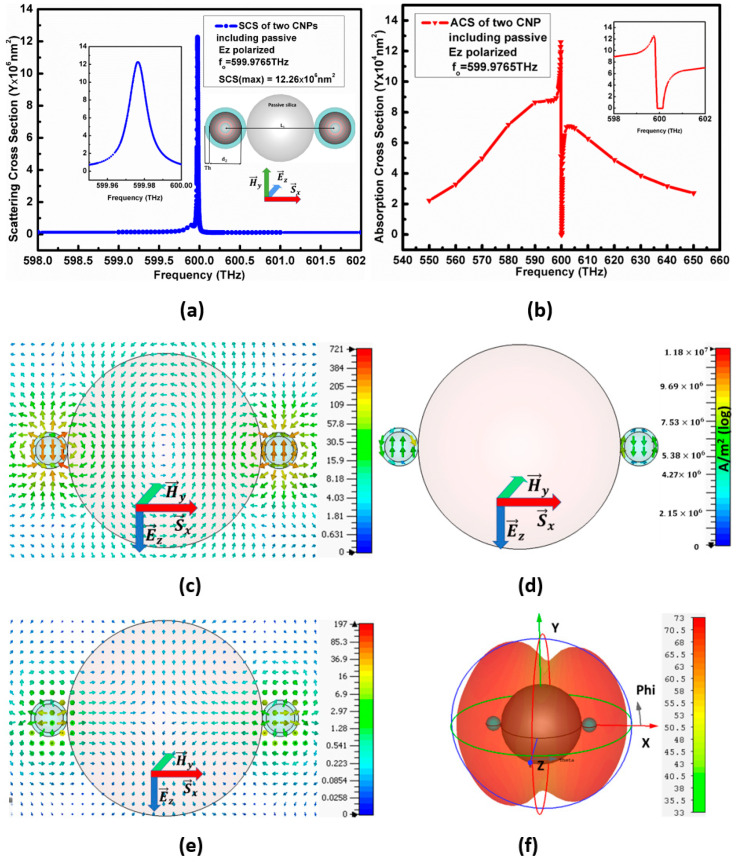
Represents (**a**) SCS peaks (**b**) ACS peak, (**c**) electric field, (**d**) electric current density, (**e**) power flow and (**f**) far-field pattern for configurations-II at resonance frequency.

The configuration II had also considered the ${\vec{E}}_{z}$ polarized plane-wave propagating along the plane of configuration that is the *x*-axis. Here, the configuration II resonates at the frequency $f_{o}=599.9765\;\mathrm{THz}$ and has maximum $SCS=12.26\times 10^{6}{\;\mathrm{nm}}^{2},$ as sketched in Figure 7a. In addition to this, the minimum ACS for configuration II is $7\times 10^{4}{\;\mathrm{nm}}^{2}$ below the background values, as shown in Figure 7b. The maximum of the electric field is 721 V/m at the resonant frequency, as shown in Figure 7c.

Figure 7 gives the variations in optical parameters when the ${\vec{E}}_{z}$ polarized plane-wave propagating along the plane of configurations excites the configuration II. The electric current density is $1.18\times 10^{7}\;A/m^{2}$, shown in Figure 7d, whereas the scattered power shown by arrows in Figure 7e reports $197\;\mathrm{VA}/m^{2}$ power flow. Here, the scattered power generates from the centers of both CNPs and flows along the *y*-axis. The far-field pattern for configuration II in Figure 7f reports $73{\;\mathrm{dBnm}}^{2}\;$ RCS. Similar to a result in Figure 5d and Figure 6f, here the far-field pattern like the pattern of the binary array, with equal amplitude and inverse phase. The flow of energy is still in the same direction as the ${\vec{E}}_{y}$ polarized plane-wave.

Table 1 gives a summarized comparison of the two basic configurations that are I and II. The first two columns compare the optical characteristics of configuration I and II while the last two columns give the comparison of configuration-II with configuration-I when the configuration II is ${\vec{E}}_{y}$- and ${\vec{E}}_{z}$-polarized. By comparing the first two columns, it is noticeable that the electric field and power flow are enhanced when a dielectric is inserted between the two A-CNPs. This consequently ensures that the constrained increase in scattering cross-section and radar cross-section, which are smaller than configuration-I, is because the dielectric behaves like a capacitive medium that stores energy and does not lower the power flow. Furthermore, the electric field of excited A-CNPs remains perpendicular when the configuration-II is excited by either an ${\vec{E}}_{x}$ or ${\vec{E}}_{y}$ polarized plane-wave. Likewise, the propagation direction of the scattered wave remains the same for all three conditions of excitation polarization discussed in the last three columns for configuration II.

## 4. Comparison of Possible 4-CNP Array Models

**Figure 8:**
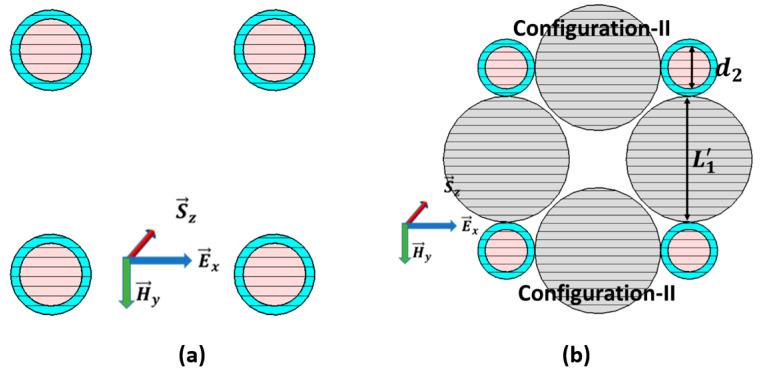
Displays schematic diagram of (**a**) array model-I with four CNPs in the free space; (**b**) array model-II comprising two units of configuration-II.

Two different array model designs, model I and II, based on configuration I and II, have been constructed and simulated to observe the enhancement of optical characteristics thoroughly, as illustrated in Figure 8a,b, respectively. The parameters of the model I are the same as deliberated for configuration I, whereas, in the case of model II, the diameter of the gain core is $d_{1}=44\;\mathrm{nm}$ and the passive diameter is $L_{1}^{\prime }=211\;\mathrm{nm},$ keeping a separation distance from the center of CNPs of 0.534λ. Model-II, shown in Figure 8b, consists of four CNPs 0.534λ separated by passive silica along the sides. In contrast, model-I, reported in Figure 8a, includes four CNPs positioned a half-wavelength apart along the sides, excluding passive silica. The ${\vec{E}}_{x}$-polarized plane-wave propagating along the *z*-axis excites the two models.

### 4.1. Excitation of Model-I by ${\vec{E}}_{x}$ Polarized Plane-Wave

**Figure 9:**
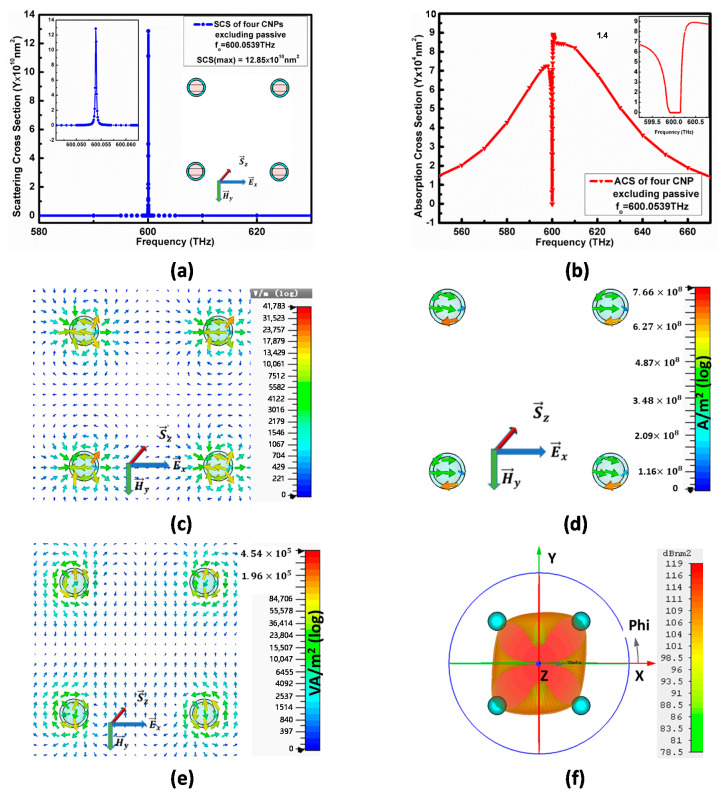
(**a**) shows schematics diagram of model-I along with SCS, while (**b**) gives its ACS. Figure 9 (**c**,**d**) explains the electric field and electric current density. Figure 9 (**e**,**f**) represents the power flow pattern and far-field pattern for model-I, respectively.

Figure 9a,b enlightens the SCS and ACS of the model I, where (a) reports the maximum $12.85\times 10^{10}{\;\mathrm{nm}}^{2}$ for model-I at the frequency $f_{o}=600.0539\;\mathrm{THz}$, whereas the minimum of ACS for model-I is $7\times 10^{4}\;{\mathrm{nm}}^{2}$ below the background values, as conveyed in (b).

Similarly, the electric field in Figure 9c for model-I is 41,783 V/m, and its current density is $7.6\times 10^{8}A/m^{2}$ at the resonance condition $\;f_{o}=600.0539\;\mathrm{THz}$.

From Figure 9e for the model I, the XY-cut plane view reports $4.54\times 10^{5}\;\mathrm{VA}/m^{2}$ power flow, where the power flow for this model generates from the centers of CNPs and is scattered in all directions. The far-field pattern in Figure 9f reports $119{\;\mathrm{dBnm}}^{2}$ RCS for model I and supports the power flow pattern.

### 4.2. Analysis of Array Model II Excited by ${\vec{E}}_{x}$ Polarized Plane-Wave

**Figure 10:**
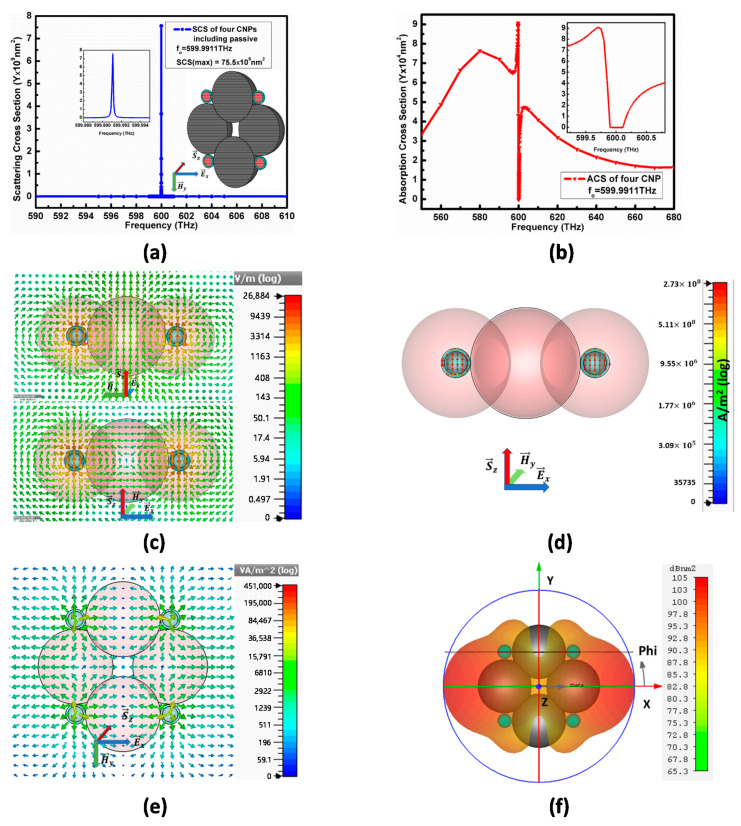
Excited model-II array: (**a**) SCS for four CNPS including passive spheres shows schematics diagram of model-II. (**b**) ACS for model-II. (**c**) E field in x-cut-plane and y-cut-plane at f = 599.9911 THz. (**d**) current distribution in y-cut-plane. (**e**) power flow in z-cut-plane, and (**f**) far-field at f = 599.9911 THz.

The ${\vec{E}}_{x}$ polarized plane-wave propagating along the *z*-axis now excites the model II comprising two units of configuration-II, separated by a similar passive size of diameter $\left(L_{1}^{\prime }\right)$, as shown in Figure 8b. Figure 10 explains the complete description of optical characteristics for this model II when it radiates after exciting it by plane-wave. The maximum SCS peak for model II in Figure 10a increases to $SCS=75.5\times 10^{9\;}{\mathrm{nm}}^{2}$ at resonance frequency $f_{o}=599.9911\;\mathrm{THz}$, which is a similar increase as for model I.

Here, the entire individual CNPs and passive dielectrics behave as a single element antenna source, where the passive dielectric provides a stable environment to the CNPs with no negative effect on the scattering cross-section.

Besides this, the minimum ACS for model II is $4\times 10^{4\;}{\mathrm{nm}}^{2}$ below the background values, as conveyed in (b). Similarly, in Figure 10c, the electric field for model II is shown from two different sides, top view in the YZ-cut plane, and side view in the ZX-cut plane, to verify the flow of the electric field along *z*-axis when the CNPs resonates. At the resonance frequency $f_{o}=599.9911\mathrm{THz},$ this array model-II reports 26,884 V/m electric field in Figure 10c. Likewise, the directions of E-fields along the *x*-axis, exterior and between the two configurations, it causes constructive coupling that enhances the E-field.

Figure 10d–f reflects the current density, power flow and far-field pattern of the model II, respectively, when it is excited by the ${\vec{E}}_{x}$-polarized plane-wave. Figure 10d signifies $2.7\times 10^{8}\;A/m^{2}$ current density, whereas the XY-cut plane view in Figure 10e reports $4.51\times 10^{5}\;\mathrm{VA}/m^{2}$ power flow at the resonance frequency for model II. The power pointing out from the center of the corner CNPs flows along the *x*-axis for model I, similar to the configuration II in all cases, as shown in Figure 10e. Both the current density and power flow enhancements are vibrant for model-II compared with configuration-II and have a similar increase as in the case of the array model I, shown in Figure 9e.

The far-field pattern in Figure 10f reports $105{\;\mathrm{dBnm}}^{2}$ RCS for model II. In comparison with configurations I and II or with a single CNP, the far-field gain for model II increases because of reducing destructive coupling in the far-field region. The far-field pattern also reports a scattering energy direction along the *x*-axis that supports the power flow direction.

It is noticeable that the direction of the scattered electric field in the entire A-CNPs and scattered radiation is rotated again for model-II. The current distribution in (d) confirms the rotation of the electric field from the *x*-axis to the *z*-axis, whereas the power flow in (e) and far-field pattern in (f) ensure the propagation of scattering radiation along the *x*-axis instead of the *z*-axis. Here, we are stating again that the increase in the electric field, current density, power flow and RCS provide evidence that the dielectric provides a capacitive behavior and stores the energy. Consequently, it reduces the losses in power flow but limits the scattering cross-section.

Table 2 gives a summarized comparison of the optical properties for model I and II with configuration I and II, respectively, when they are ${\vec{E}}_{x}$-polarized. The enhancement in all optical characteristics is clearly shown for model I and II in column III and column V, respectively.

## 5. Extended Array of Model-II to 12 A-CNPs

The extended form of model-II includes the periodic arrangement of 12 CNPs, which has a similar set of parameters as CNPs but different separation between the CNPs occupied by the passive spheres. This extended array is observed for different passive separation size to obtain the maximum optical characteristics. We find the maximum optical characteristics for the passive material of the size $L_{1}^{\prime }=307.9\;\mathrm{nm}$ to maintain 0. 727*λ* spacing between the two CNPs.

**Figure 11:**
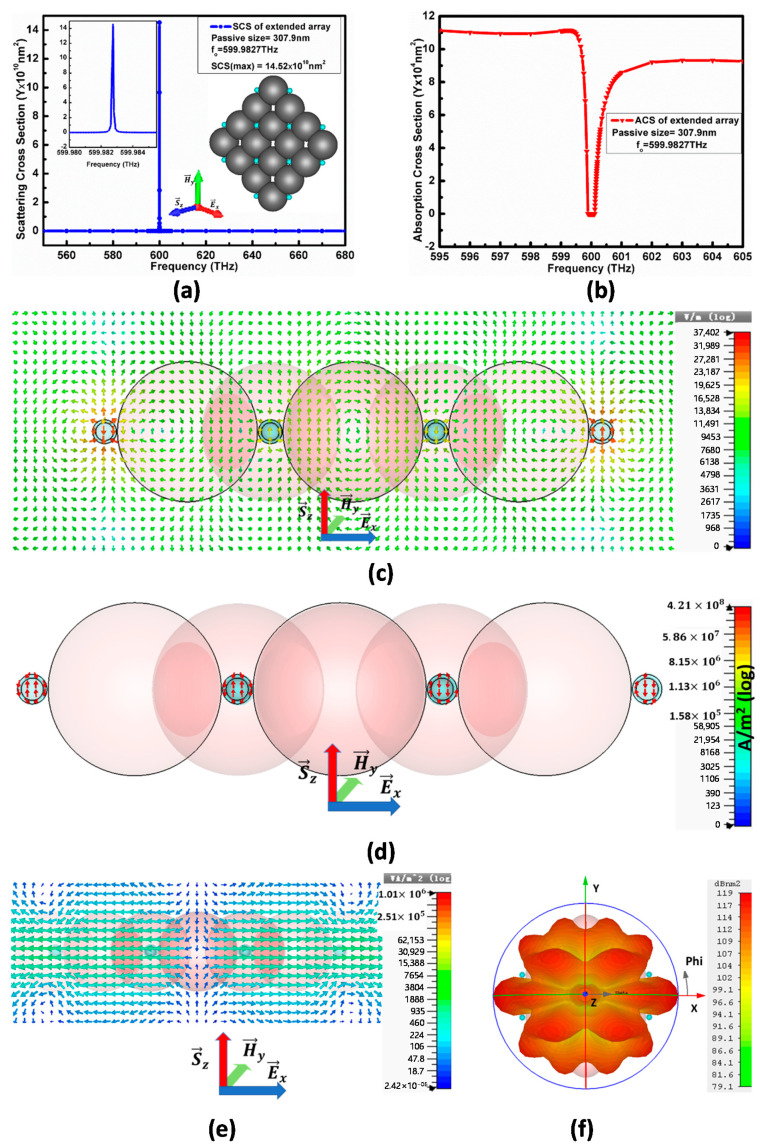
(**a**) denotes SCS peak for extended array model and (**b**) represents its ACS at resonance Figure 11. (**c**) denotes electric field pattern from top view while (**d**) shows its current density. Similarly, (**e**) gives power flow spectrum and (**f**) far-field pattern of extended array model at resonance state.

The extended array has a similar diameter of active core and thickness of CNPs to the model-II but has an $L_{1}^{\prime }=307.9\;\mathrm{nm}$ diameter of passive separation between CNPs, to maintain 0.727*λ* spacing between the two CNPs, as shown on the right side with an SCS peak in Figure 11a. When the ${\vec{E}}_{x}$ polarized plane-wave propagating in the ${\vec{S}}_{z}$ direction excites this extended array, it resonates at the frequency $f_{o}=599.9827\;\mathrm{THz}$ and gives a maximum SCS peak as $SCS=14.52\times 10^{10}\;{\mathrm{nm}}^{2}$, whereas the minimum of ACS in Figure 11b at the same resonance condition is $10\times 10^{4\;}{\mathrm{nm}}^{2}$ below the background values. Therefore, the maximum absorption occurs at resonance states; in other words, we can state that more energy is stored by the array and, consequently, the SCS reduces. The inadequate increase in SCS is due to the compact structure that increases coupling in the near-field. Similarly, the ACS results also support the argument of increasing coupling.

Figure 11c–f reports the electric field pattern, current density, power flow and the far-field pattern at the same resonance condition as for the extended array. It shows a $3.74\times 10^{4\;}V/m$ electric field, $4.21\times 10^{8}\;A/m^{2}$ electric current density and $1.01\times 10^{6\;}\mathrm{VA}/m^{2}$ power flow.

The current density arrows in Figure 11d are aligned vertically and have no horizontal components along the *x*-axis, whereas the current in each two CNPs is out of phase for the two nearby CNPs, hence cancelling their effect, which consequently reduces the flow of the electric field along the *z*-axis.

From the power flow pattern in Figure 11e, the arrow indicates the power generation from the center of all CNPs, directed along the positive and negative *x*-axis and resulting in a multimode scattering perpendicular to the incident plane-wave direction. Similarly, the far-field pattern presented in Figure 11f reports the maximum $119\;{\mathrm{dBnm}}^{2}$ RCS along the *x*-axis in a multimode form supporting the arguments of power flow pattern and current density.

Furthermore, the scattered electric field in the entire A-CNPs shown in Figure 11c is rotated by 90° to the excitation electric field in a similar way as in the case of configuration II (two A-CNPs case) and in the case of model II (4 A-CNPs case). The current distribution in (d) supports and confirms the rotation of this scattered electric field.

### Variation in Scattering Direction and Directivity at Nearby Frequencies

The directivity and scattering direction, interestingly, change at adjacent frequencies, from a multi-directional beam to a directional beam. Even at smaller frequencies, variations that are 0.01 THz below and 0.008 THz above the resonance frequency of the multimode shown in Figure 11f convert into a directive scattering beam along the plane-wave direction.

**Figure 12:**
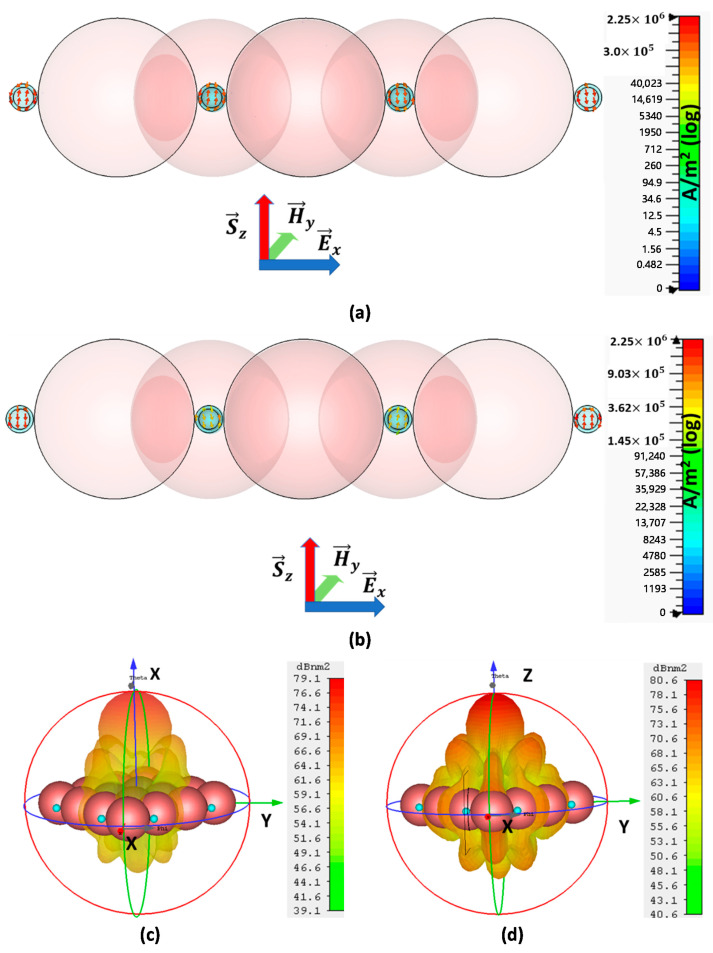
Current distribution and far field of the extended array of model-II at nearby resonance frequency: (**a**) current density below resonance frequency (at f=599.970 THz), (**b**) current density above resonance frequency (at f=599.9911 THz), (**c**) far-field pattern at lower frequency f=599.970 THz and (**d**) the far-field pattern at higher frequency f=599.9911 THz) for extended array.

Figure 12a,b shows current densities at similar below-resonance frequencies, i.e., $f_{1}=599.9700\;\mathrm{THz},$ and similar above-resonance frequencies, i.e., $f_{2}=599.9911\;\mathrm{THz}$.

The current density arrows in Figure 12a,b are not exactly in vertical directions but have both vertical and horizontal components for each CNP. Furthermore, the vertical components of all the CNPs in a row are out of phase because the current of the two CNPs is along the positive *z*-axis and the current of the other two CNPs is along the negative *z*-axis. Hence, vertical components will get cancelled and, consequently, there will be no flow of electric field along the *z*-axis.

Despite this, the horizontal components of currents along the *x*-axis of nearby CNPs are supported in the same direction, which results in the flow of electric field along the *x*-axis. Accordingly, it compels the beam formation of scattering along the *z*-axis.

The far-field pattern in Figure 12c,d reports a higher directional beam along the *z*-axis at slightly different frequencies from the resonant frequency. Moreover, they support the above arguments for current density and electric field. The maximum RCS reported in Figure 12c,d is $79.1\;{\mathrm{dBnm}}^{2}$ at 599.970 THz and $80.6{\;\mathrm{dBnm}}^{2}$ at 599.9911 THz, respectively, for the same array model.

## 6. Conclusions

Generally, in the nano-particle array model, instability issues persist. The article suggests the addition of passive dielectric (SiO_2_) between antenna array elements (A-CNPs) to enhance stability and reduce agility during the experimental fabrication of the array model on a substrate. This paper presents a comparison of two different configurations of active core-shell nano-particle antennas, as well as their two extended array models for the enhancement of optical characteristics in a stable environment. A non-conventional behavior is observed for the extended array model at a resonance frequency that shows a strong multi-polar beam with a 90° rotated electric field as well as a rotated scattered plane-wave with an incident plane-wave. Likewise, its behavior is conventional at similar frequencies, i.e., directive at nearby frequencies and 0.01 THz below the resonance frequency and 0.008 THz above the resonance frequency. The study thoroughly observes enhancement in the electric field, radiated power, scattering cross-section, absorption cross-section and far-field gain in detail for the proposed array configuration and its extended array model in the presence of a passive dielectric. Analysis of the results revealed that the array model comprising a passive SiO_2_ between the two A-CNPs is compatible with the array model excluding a passive one between A-CNPs. The analysis demonstrates that the configuration of active spherical coated nanoparticles separated by a passive dielectric can improve the optical characteristics and is better than other configurations for nano-amplifier and nano-sensor applications.

## Figures and Tables

**Table 1:** Comparison of optical properties for the two basic configurations I and II.

Parameters	Array of Two A-CNPs Excluding Dielectric *f*_0_ = 600.0195 THz	Array of Two A-CNPs Including Dielectric *f*_0_ = 599.9953 THz	Array of Two A-CNPs Including Dielectric (${\vec{E}}_{y}$ Polarized) *f*_0_ = 599.9924 THz	Array of Two A-CNPs Including Dielectric (${\vec{E}}_{z}$ Polarized) *f*_0_ = 599.9765 THz
SCS (Max)	$127.79\times 10^{9}{\;\mathrm{nm}}^{2}$	$11.53\times 10^{9\;}{\mathrm{nm}}^{2}$	$94.4\times 10^{6}\;{\mathrm{nm}}^{2}$	$12.26\times 10^{6}{\;\mathrm{nm}}^{2}$
RCS (Far Field)	$116\;{\mathrm{Bnm}}^{2}$	$103\;{\mathrm{Bnm}}^{2}$	$82.2\;{\mathrm{Bnm}}^{2}$	$73\;{\mathrm{Bnm}}^{2}$
E field (Near field)	10,008 V/m	20,493 V/m	2575 V/m	721 V/m
Power Flow	$3.1571\times 10^{4}\;\mathrm{VA}/m^{2}$	$1.66\times 10^{5}\;\mathrm{VA}/m^{2}$	$2.236\times 10^{3}\;\mathrm{VA}/m^{2}$	$1.97\times 10^{2}\;\mathrm{VA}/m^{2}$
Rotation of electric field	0°	90°	90°	0°

**Table 2:** Comparison of optical properties for the two models (I and II) with the two configurations (I and II).

Parameters	Array of Two A-CNPs Excluding Dielectric (${\vec{E}}_{x}$ Polarized) *f*_0_ = 600.0195 THz	Array of Four A-CNPs Excluding Dielectric (${\vec{E}}_{x}$ Polarized) *f*_0_ = 600.0539 THz	Array of Two A-CNPs Including Dielectric (${\vec{E}}_{x}$ Polarized) *f*_0_ = 599.9953 THz	Array of Four A-CNPs Including Dielectric (${\vec{E}}_{x}$ Polarized) *f*_0_ = 599.9911 THz
SCS (Max)	$127.79\times 10^{9}{\;\mathrm{nm}}^{2}$	$12.85\times 10^{10}\;{\mathrm{nm}}^{2}$	$11.53\times 10^{9}{\;\mathrm{nm}}^{2}$	$75.5\times 10^{9}{\;\mathrm{nm}}^{2}$
RCS(Far Field)	$116\;{\mathrm{Bnm}}^{2}$	$119\;{\mathrm{Bnm}}^{2}$	$103\;{\mathrm{Bnm}}^{2}$	$105\;{\mathrm{Bnm}}^{2}$
E field (Near field)	10,008 V/m	41,743 V/m	20,493 V/m	26,884 V/m
Power Flow	$3.1571\times 10^{4}\;\mathrm{VA}/m^{2}$	$4.54\times 10^{5}\;\mathrm{VA}/m^{2}$	$1.66\times 10^{5}\;\mathrm{VA}/m^{2}$	$4.51\times 10^{5}\;\mathrm{VA}/m^{2}$
Rotation of electric field	0°	0°	90°	90°

## Data Availability

The data presented in this study are available on request from the corresponding author.
